# The necessity for full ventricular coverage with noncontrast T1 mapping in hypertrophic cardiomyopathy

**DOI:** 10.1186/1532-429X-15-S1-E1

**Published:** 2013-01-30

**Authors:** Ethan Rowin, Martin Maron

**Affiliations:** 1 Hypertrophic Cardiomyopathy Center, Tufts Medical Center, Boston, MA, USA

## Background

Hypertrophic cardiomyopathy (HCM) is a genetic heart disease characterized by diverse phenotypic expression. T1 mapping is a novel CMR imaging sequence aimed at characterizing abnormal myocardial substrate. Current protocols obtain a mean T1 value derived from a single mid-LV short axis slice (single slice protocol) or three short-axis locations: apical, mid-LV and basal (three slice protocol). However, given the heterogeneity of disease expression within HCM, it is unclear if T1 values obtained from these limited portions of the LV chamber are representative of values derived from the entire LV myocardium (global).

## Methods

CMR imaging with a noncontrast T1 sequence using a Shortened Modified Look-Locker Inversion recovery (ShMOLLI) protocol was performed in 17 consecutive HCM patients (57 ± 16 years old; 82% male). T1 maps of sequential 10 mm short axis slices from the atrioventricular ring to the apex were acquired in each patient and a mean T1 value was derived for each short-axis slice and then summed to generate a global LV T1 value, which were compared to mean T1 values derived from a single and three slice protocol.

## Results

The mean global LV T1 value was 864 ± 52 ms (range 731-998 ms), 850 ms ± 59 (range 741-942 ms) for the single segment protocol and 869 ms ± 57 (range 753-971 ms) for the 3 segment protocol. Throughout the entire LV chamber, there was significant variation among T1 values for each short-axis slice (p<0.001), with mean T1 values significantly higher in the basal and apical levels compared to the mid-LV level (875 ± 56 vs 864 ± 65 vs 855 ± 52 ms; p=0.01). T1 values using a single slice protocol were significantly shorter then global LV T1 values (850 ± 59 vs 864 ± 52; p=0.05), and, while not statistically significant, T1 values were higher with a three slice protocol than compared to global LV values (869 ± 57 vs 864 ± 52; p=0.14).

## Conclusions

In a morphologically heterogeneous disease such as HCM, significant variations exist among T1 values across each short-axis slice of the LV myocardium. In addition, T1 values obtained using a single mid-LV short axis slice were significantly less than global T1 values. These findings support the necessity to obtain mean T1 values derived from the entire LV myocardium in patients with HCM.

## Funding

Not applicable

